# The Fragile State of Industrial Agriculture: Estimating Crop Yield Reductions in a Global Catastrophic Infrastructure Loss Scenario

**DOI:** 10.1002/gch2.202300206

**Published:** 2023-12-20

**Authors:** Jessica Moersdorf, Morgan Rivers, David Denkenberger, Lutz Breuer, Florian Ulrich Jehn

**Affiliations:** ^1^ Institute for Landscape Ecology and Resources Management (ILR) Research Centre for BioSystems Land Use and Nutrition (iFZ) Justus Liebig University Giessen Heinrich‐Buff‐Ring 26 35390 Giessen Germany; ^2^ Alliance to Feed the Earth in Disasters (ALLFED) Lafayette CO 80026 USA; ^3^ Department of Mechanical Engineering University of Canterbury Christchurch Canterbury 8041 New Zealand; ^4^ Centre for International Development and Environmental Research (ZEU) Justus Liebig University Giessen Senckenbergstraße 3 35392 Giessen Germany

**Keywords:** electrical grid, food security, global catastrophic infrastructure loss, global catastrophic risks, solar storm

## Abstract

Modern civilization relies on a complex, globally interconnected industrial agriculture system to produce food. Its unprecedented yields hinge on external inputs like machinery, fertilizers, and pesticides, rendering it vulnerable to disruptions in production and international trade. Such a disruption could be caused by large‐scale damage to the electrical grid. Solar storms, nuclear detonations in the upper atmosphere, pandemics, or cyber‐attacks, could cause this severe damage to electrical infrastructure. To assess the impact of such a global catastrophic infrastructure loss on major food crops (corn, rice, soybean, wheat), we employ a generalized linear model. The predictions show a crop‐specific yield reduction between 15% and 37% in phase 1, the year after the catastrophe, assuming rationed use of fertilizers, pesticides, and fuel stocks. In phase 2, when all stocks are depleted, yields decrease by 35%–48%. Soybean is less affected in phase 1, while all crops experience strong declines in phase 2. Europe, North and South America, and parts of India, China, and Indonesia face major yield reductions, potentially up to 75%, while most African countries are less affected. These findings underscore the necessity for preparation by highlighting the vulnerability of the food system.

## Graphical abstract

Modern farming, dependent on machinery, fertilizer and pesticides, is at risk from electrical grid disruptions due to various catastrophes. Yields may drop 15%–37% in the first year and 35%–48% after industrial inputs run out, varying by crop. Europe, the Americas, and parts of Asia can see up to 75% yield reductions. Preparation is crucial.

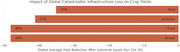

## 1 Introduction

Global food security is at risk from major disruptions.^[^ ^1^ ^]^ Over time humanity has built an increasingly complex food system, with global trade connecting food producers and consumers. The hope being that this would make the food system more resilient to disruptions.^[^ ^1^ ^]^ This seems to have worked partially and many systemic risk studies describe the food system as “robust, yet fragile”, meaning that it is able to buffer smaller shocks more easily, but has become more vulnerable to major ones.^[^ ^2^, ^3^, ^4^ ^]^ This increased vulnerability to major shocks is grounded in the finding that many of the globally traded goods like virtual water,^[^ ^5^ ^]^ food commodities,^[^ ^3^ ^]^ or fertilizer^[^ ^6^ ^]^ are concentrated into few, but major players like the United States. If these major players stopped trading, the whole system would be in danger, due to cascading failure.^[^ ^4^, ^7^, ^8^ ^]^ These ideas from systemic risk have been picked up in the study of global catastrophic risks as well.^[^ ^9^, ^10^ ^]^ The food system is not only increasingly vulnerable to major disruptions like multiple breadbasket failures but^[^ ^11^, ^12^, ^13^ ^]^ there are also a variety of global catastrophic risks, which could impact the food system. These include asteroid/comet impacts, volcanic eruptions, ecosystem collapse, nuclear war, and termination shock caused by solar radiation management.^[^ ^14^, ^15^, ^16^ ^]^ This is concerning as society is highly dependent on modern agriculture. It enables most of the population to occupy themselves with tasks beyond food production.^[^ ^17^, ^18^ ^]^

Agriculture facilitated the emergence of complex societies all around the world^[^ ^19^ ^]^ and is needed to sustain it. Agricultural practices developed simultaneously in multiple different cultures, but yields were low and crop production labor intensive: despite its merits, food production in agricultural societies still required the involvement of most of the population to feed everyone. It was not until the rise of modern technology which allowed the harnessing of energy from fossil fuels and its introduction into agriculture in the shape of machinery, artificial fertilizer, and pesticides during the twentieth century that human populations could grow substantially and employ a decreasing fraction of society in agriculture. This stark increase was supported by an expansion of cropland^[^ ^20^ ^]^ and by substantially decreasing the number of human work hours required to produce one ton of grain.^[^ ^19^ ^]^ The surplus in food and energy production can only be maintained through high external inputs into the production system in the form of machinery, fertilizers, and pesticides.^[^ ^21^ ^]^ The significance of outside influences varies from one country to another because there is no single standard agricultural production system, and there are significant variations between countries and global regions. Nevertheless, even in countries with lower reliance on industry, they are integrated into the increasingly interconnected global system, which means they are likely to be affected by the repercussions of widespread failures.^[^ ^1^, ^2^ ^]^ These characteristics, marked by a strong dependence on industry and global interconnectivity, have emerged in the past century and have rapidly spread, bringing about profound and enduring societal transformations.

In light of this, it becomes evident that global society depends on a reliable food supply and this food supply is only stable as long as the constant flow of inputs like fertilizer is possible. However, significant disturbances have the potential to unsettle the production of those inputs, as well as the food system itself. While extensive research has been conducted on regional hazards,^[^ ^22^, ^23^, ^24^ ^]^ as well as global, long‐term disruptions such as environmental impacts,^[^ ^25^, ^26^ ^]^ challenges related to climate change,^[^ ^12^, ^27^, ^28^, ^29^ ^]^ and the decreasing rates of yield increase,^[^ ^30^ ^]^ little is known about sudden, global events. On the effects of the disruptions of global trade and industrial infrastructure on agriculture, only exploratory research exists.^[^ ^31^ ^]^ While such events are seen as unlikely, the COVID‐19 pandemic has demonstrated that events deemed highly unlikely can still occur at any given time and has exposed the lack of preparedness in most countries.^[^ ^32^, ^33^ ^]^

This paper models the anticipated change in agricultural yield in such a sudden and global disruption of the infrastructure needed to sustain the food system, a global catastrophic infrastructure loss scenario. The underlying premise of all possible causes for global catastrophic infrastructure loss is a global‐scale disruption of the electrical grid. Given the widespread dependence of global industry and society on electricity, a global electrical failure would essentially bring most industries and machinery to a standstill. The four main potential causes for global catastrophic infrastructure loss include:

- High Altitude Electromagnetic Pulses (HEMP) result from nuclear detonations high in the atmosphere. They cause no immediate harm to humans but can almost instantly damage electronics. Detonating a nuclear warhead emits gamma rays that interact with the atmosphere, creating an intense electromagnetic pulse (EMP) spreading at light speed. The disruptive EMP causes electronics to suffer overvoltage, like a more powerful lightning strike.^[^ ^34^ ^]^ The affected area depends on the detonation's power and altitude; one detonation could affect the entire contiguous United States.^[^ ^34^ ^]^ Multiple warheads during a nuclear conflict could lead to a global catastrophe. Recovery would likely be difficult, as critical infrastructure like large power transformers are often highly customized and currently need 12–24 months for production.^[^ ^35^ ^]^
- A similar risk is posed by solar storms. Solar activity during storms can present itself in the form of solar flares, coronal mass ejections, or both. Solar flares are bursts of x‐ and gamma rays and extreme ultraviolet radiation that can disrupt communication technology.^[^ ^36^, ^37^, ^38^ ^]^ Other research emphasizes the effect of coronal mass ejections on the American power grid.^[^ ^39^ ^]^ This type of solar activity releases supercharged plasma particles toward the earth, creating a geomagnetic storm that acts like a natural EMP toward the electrical grid with potentially devastating consequences.^[^ ^35^, ^36^, ^40^ ^]^ Like HEMPs, coronal mass ejections can permanently damage large power transformers and thus potentially cause power outages lasting for years.^[^ ^35^ ^]^
- Globally coordinated cyber‐attacks on many electrical grids or critical industrial infrastructures pose a threat on a global catastrophic scale. Among the various systems under attack, power generation is a prime target for these cyber‐attacks.^[^ ^41^ ^]^ Until now, such attacks have been relatively limited in scope, but there is concern that more advanced and motivated actors could cause significant damage and disruption to these essential systems on a larger scale.
- An extreme pandemic could cause people to be too fearful to report to work in critical industries, resulting in a collapse of the power grid and other infrastructure, as maintenance ceases.^[^ ^42^ ^]^ This pandemic would have to be considerably more deadly than COVID‐19 to create such an effect.

All this highlights that it is important to increase the stability of the global food system. Resilience efforts for the food production system vary depending on the type of catastrophe. For sun‐blocking scenarios like a supervolcanic eruption this includes the exploration and preparation of resilient foods such as single‐cell protein from natural gas,^[^ ^43^ ^]^ hydrogen,^[^ ^44^ ^]^ sugar from wood,^[^ ^45^ ^]^ greenhouses,^[^ ^46^ ^]^ or seaweed.^[^ ^47^ ^]^ More traditional resilience measures include food storage, diversification of agricultural practices, crop insurance, or regulations of the agricultural market.^[^ ^1^, ^15^ ^]^ Most of these solutions, however, depend on industrial infrastructure in one way or another or assume that only a smaller subsection of global food production is impacted. Therefore, for global catastrophic infrastructure loss scenarios, the adaptation of classical agricultural practices is the main method to ensure food security. Earlier work has suggested that this could revert agricultural yield to preindustrial levels.^[^ ^31^ ^]^

This research aims to offer a more accurate and geographically detailed global‐scale assessment of the potential impact of catastrophic infrastructure loss on crop production.

Based on a multiple regression model using spatial predictors, we project yields for a worst‐case scenario to understand the effects of a disturbance of industrial infrastructure on modern agriculture.

## 2 Experimental Section

### 2.1 Selection of Model Crops and Influencing Factors

This study focused on modeling the yields of four fundamental crops: wheat, corn, rice, and soybeans. These crops were deliberately chosen due to their pivotal role as staple foods, as determined by analyzing data from FAOSTAT, which includes their annual production quantities and harvested areas. Together, these four crops account for a substantial 57% of the calories and 61% of the protein in the human diet.^[^ ^48^ ^]^ By focusing on those crops, it had a good proxy for the food system overall. This approach allows to represent the food system comprehensively without the need to examine the vast array of food commodities that exist.

On a global scale, wheat and rice is the primary staples in the human diet.^[^ ^48^, ^49^, ^50^ ^]^ Meanwhile, corn and soybean production are also directed toward livestock and aquaculture feed in large quantities.^[^ ^51^, ^52^ ^]^ In the event of a global catastrophic infrastructure loss, both corn and soybean crops had large potential because their production could be redirected for human consumption. In addition, soybeans could play a pivotal role in maintaining nitrogen availability in the soil in the absence of industrial fertilizers, as they can fix nitrogen from the air.

Crop yield was influenced by a variety of factors, like crop variety, nutrients, water, climate, mechanization, seed availability, knowledge of farmers, pests, and diseases.^[^ ^30^, ^53^, ^54^ ^]^ The yield influencing factors used as model inputs for the analysis were chosen based on two selection criteria:

1. We identified key factors that played a pivotal role in the progress of agriculture from preindustrial to modern times. Consequently, mechanization, fertilizer, irrigation, and pesticides were selected in conjunction with enhanced crop varieties.^[^ ^19^, ^21^, ^55^ ^]^
2. All factors with inadequate data availability that fell short of the spatial data resolution of five arcminutes at a global scale were excluded. Therefore, the improved varieties had to be excluded in the second step due to insufficient data availability. This exclusion of relevant variables likely leads to an underestimation of yield loss, but could not be avoided as no global, high‐quality data was available.

The availability of the factors listed above was directly dependent on the management decisions of the farmer. However, there were also influential elements like climatic conditions that could not be managed. To control for their impact on crop yield, three climatic variables representing thermal, moisture, and soil conditions were considered in the analysis.

### 2.2 Spatial Data

Global spatial datasets were sourced for each factor as well as for yields under current conditions. Datasets were selected at five arcminutes resolution when available or downsampled to this resolution (**Table** **1**; additional information can be found in Description_input_data.pdf in the repository of this paper^[^ ^56^ ^]^).

**Table 1:** Datasets Used for Calibrating the Generalized Linear Model and Simulating Loss of Industry Scenario Conditions.

Dataset	Definition	Spatial resolution	Time Period	Source	Available online
SPAM	yield (kg ha^−1^), harvested area (ha/cell)	5 arcmin	22010	Yu et al. (2020)^[^ ^57^ ^]^	https://doi.org/10.7910/DVN/PRFF8V
GAEZ v4 AEZ Factors	thermal regime class, moisture regime class, soil/terrain‐related class	5 arcmin,5 arcmin,30 arcsec	22010	Fischer (2021)^[^ ^58^ ^]^	https://gaez.fao.org/pages/data‐viewer
PEST‐CHEMGRIDS	application rate (kg/ha) of 20 active ingredients for 10 dominant crops and four aggregated crop classes	5 arcmin	22015	Maggi et al. (2019)^[^ ^59^ ^]^	https://doi.org/10.7927/weq9‐pv30
Global Map of Irrigation Areas – Version 5	area equipped for irrigation (% of total area)	5 arcmin	22005	Siebert et al. (2013)^[^ ^60^ ^]^	https://data.apps.fao.org/map/catalog/srv/api/records/f79213a0‐88fd‐11da‐a88f‐000d939bc5d8
AQUASTAT – FAO's Global Information System on Water and Agriculture	Area (1000 hectares) equipped for: Irrigation (Equipped Lowland Areas, Spate Irrigation, Total) Full control irrigation (Surface, Sprinkler, Localized, Total, Actually Irrigated) Power irrigation	Country level	Around mid‐2010s	FAO (2019)^[^ ^61^ ^]^	http://fao.org/aquastat/statistics/query/index.html?lang=en
Gridded nitrogen and phosphorus fertilizer use	N and P application rate (g/m^2^)	0.5◦degree	11900‐2013	Lu and TIan (2016)^[^ ^62^ ^]^	https://doi.pangaea.de/10.1594/PANGAEA.863323
Global gridded dataset of manure nitrogen production and application	N manure application (kg/km^2^)	5 arcmin	11860‐2014	Zhang et al. (2017)^[^ ^63^ ^]^	https://doi.pangaea.de/10.1594/PANGAEA.871980
A global gridded data set on tillage (V. 1.1)	six tillage systems (dominant system/cell)	5 arcmin	Aaround 2005	Porwollik et al. (2019)^[^ ^64^ ^]^	https://doi.org/10.5880/PIK.2019.009

The N manure and N fertilizer application rate datasets from Table 1 were summed up into a combined variable N total, as the analysis was only concerned with the effect reduced N input had on yield and not with the effect of N input from different sources. Moreover, it was taken as a measure to reduce the number of variables and possible multicollinearity between them. Nitrogen management could not be considered due to a lack of suitable, global data. The data pre‐processing described in the next section was done before this merge, to be able to detect outliers.

Mechanization was the only selected factor that required the use of a proxy as no spatially explicit data on the degree of mechanization in agriculture was available. The “global gridded data set on tillage (V. 1.1.)”^[^ ^64^ ^]^ was used as a surrogate to determine if an area was farmed with motorized agricultural machinery or based on human and animal draft power. A large factor in the classification of tillage systems was the involvement of heavy machinery as it facilitates plowing soils in greater depth. Hence, it was possible to use the tillage systems as a proxy to determine, which systems rely on machinery for tilling and that do not. Other farm activities were assumed such as sowing and harvesting were also carried out with machinery if tilling was mechanized. Therefore, the tillage systems were reclassified into either 0  = non‐mechanized or 1 = mechanized. Conservation agriculture was classified as mechanized even though tillage was reduced to almost zero because currently conservation agriculture was most widely adopted in North and South America and Australia^[^ ^65^ ^]^ where agriculture tends to be mostly mechanized.

Misalignment between input datasets had a significant impact on model accuracy. If the spatial distribution of values did not match across datasets, it could led to a misrepresentation of the relationship between the variables under study. However, this issue was mitigated by using large datasets to ensure a sufficient overlap for accurate relationship mapping.

### 2.3 Preprocessing and Statistical Yield Modeling

Before fitting the model, it pre‐processed the data to allow for a robust statistical analysis. The following operations were carried out for each crop individually:

- The values for crop yield in kg per hectare in each cell represent a varying portion of the specific crop's harvested area ranging from 0.1 to 19344.3 ha. This large range in crop area per cell size could influence the results of the analysis, as it gives each cell the same weight, independent of the actual agricultural area in the cell. Therefore, all rows containing values for harvested areas below 100 ha were removed. This operation led to the deletion of 44%–72% of all data points (depending on the crop, as do all following ranges shown). However, these cells contributed only between 1.6% and 3.2% of the total global crop production summed up over the total crop‐specific harvested area and thus did not play an important part in global food security.
- Subsequently, missing values in the remaining datasets were addressed. Particularly the pesticides and mechanization data contained missing values. Gap filling of missing data, e.g., through interpolation, was not possible, as there was no established dependence of pesticides and mechanization on the other variables, so these data points were removed. In the N fertilizer column, missing values amounted to 1%–2.3% of total data points. The temperature, the moisture regime, and the soil/terrain‐related columns also had missing data points in the range of 1.6%–2.2%. Cells with missing data for both data sets were treated with the forward‐filling method (carrying forward the last observed value).

N fertilizer, the manure, the pesticides, and the yield contained implausible values. To prevent extreme outliers from skewing the relationship, all data with values above the 99.9th percentile for N fertilizer, manure (99th percentile), N total, pesticides, and yield were removed. Given the distribution of the remaining values and the values commonly reported in the literature, these data points were more likely to be errors in the input datasets than real information characterizing the relationship between yield and input factors. Even though there was reason to assume that more values on both ends of the scale, albeit feasible, could be attributed to calculation errors or relics of the downsampling approach, this could not be validated and therefore, it was refrained from excluding more values. Additional information on the data cleaning process and the effect of each operation on the metrics of the datasets can be found in reports/Report_descriptions.pdf and reports/Descriptive_statistics.xlsx in the repository of this paper^[^ ^56^ ^]^).

In the next step, for any multicollinearity present in the data was checked. It can be detected by calculating the variance inflation factor^[^ ^66^ ^]^ for each predictor. The literature contained different threshold values for when the VIF indicated serious multicollinearity. The most prominent thresholds were specified as everything above five,^[^ ^67^ ^]^ or as values above ten^[^ ^68^ ^]^ constitute the need for action. However, the VIF did not work well for categorical variables if they had multiple levels. So instead, it compute the generalized variance inflation factor (GVIF).^[^ ^69^ ^]^ To make it comparable across predictors with a differing number of levels, Fox and Monette (1992) suggest using $GVIF^{\frac{1}{2\times Df}}$ with Df being equal to the number of levels in each variable. Squaring this value yields the regular variance inflation factor for predictors with one level so that the variance inflation factor thresholds could be applied. The squared $GVIF^{\frac{1}{2\times Df}}$ did not indicate any multicollinearity among the variables for any crop (see the Model_VIF sheet in reports/Model_results.xlsx in the repository of this paper^[^ ^56^ ^]^).

Multicollinearity, arising from the inclusion of both nitrogen and phosphorus fertilizer application rates, had a noticeable impact on model results. As these fertilizers are often applied together, the decision was made to use nitrogen application as a proxy for nutrient input and exclude phosphorus application to mitigate multicollinearity.

As it is harder to maintain agricultural production in very cold, hot, dry, or wet climates, an uneven distribution of observations among the levels in the thermal and moisture regime classes was detected. For the thermal regime, the differences were particularly stark as the coldest three climate classes count with a very low number of observations. A highly uneven distribution of observations could lead the model to misjudge the significance of a predictor. To resolve the issue, the Temperate cool, Boreal, and Arctic regimes were aggregated. The uneven distribution of observations in the moisture regime was addressed by fusing the two lowest (M1 and M2) and the two highest levels (M6 and M7) into one new level each: M2 = Length of Growing Period <120 days and M6 = Length of Growing Period 270+ days. These merges do not reflect the best combinations for each crop. The wheat model, for example, could have benefited from combining levels T1 and T2. However, we refrained from performing different merges for each crop to ensure comparability between the crops.

Adding the variables to the model consecutively did not show any abnormalities in the standard errors or the p values. Therefore, sufficient data quality for the following analysis was estimated.

A split‐sample approach was applied to calibrate and validate the model. Prior to fitting the model, 20% of the pre‐processed data were randomly selected. This sample was used for validation while the model was calibrated on the remaining 80% of the data points.

As the dependent variable can not assume negative values, the distribution of the data points was strongly right‐skewed for all crops and the residuals were non‐normally distributed, so the assumptions for a classic multiple regression on a normal distribution were violated. Therefore, a generalized linear model based on a gamma distribution was fitted to the data. The link function was assumed to be the natural logarithm, as the data showed a normal distribution at the logarithmic scale. The model was specified as followed:

(1) $$Y\;∼\;Gamma\left(shape,\;scale\right)$$

where *Y* is the response variable that follows a gamma distribution, shape is the shape parameter of the gamma distribution (*α* >0) and scale is the scale parameter of the gamma distribution (*β* >0). The expected value (mean) of the response variable (*Y*) µ can be written as an expression of shape and scale

(2) μ=shape×scale

The log link connects µ to the linear predictor

(3) $$g\;\left(\mu \right)=\ln\;\left(\mu \right)=\eta =\beta_{0}+\beta_{1}\times x_{1}+\beta_{2}\times x_{2}+\text{…}+\beta_{p}\times x_{p}$$

where *β*_0_, *β*_1_, *β*_2_, …, *β*_p_ are the model coefficients (parameters to be estimated), *x*_1_, *x*_2_, …, *x*_p_ are the predictor variables and p is the number of predictor variables.

The model was fitted with a simple linear relationship and no interactions. The categorical variables were coded as dummies. To assess model fit, McFadden's *ρ* ^2^ was used, which is an alternative for *R* ^2^ for non‐normally distributed data. The significance level was set at *α* = 5%.

### 2.4 Yield Prediction Scenarios

Crop yields were projected under a worst‐case scenario where the industry suffers significant losses, employing a generalized linear model. This assumes a global catastrophe that disrupts power supply, leading to the inhibition of industrial activities, communication, transportation, and other electricity‐dependent services. However, it was presumed that transportation remains feasible to a certain extent, allowing farmers to receive necessary inputs and food distribution to continue.^[^ ^70^, ^71^ ^]^ While the triggering event was expected to occur suddenly, the impact on agricultural production was likely mitigated by existing stocks of inputs in storage. Consequently, the aftermath of the catastrophe was divided into two phases: phase 1 encompasses the initial year, during which stocks were still available, while phase 2 commences in the second year when stocks were depleted, and the consequences of losing electrical infrastructure manifest in their entirety. The datasets used to calibrate the model's independent variables were adjusted for predictions based on the assumptions of either phase 1 or phase 2.

#### 2.4.1 Phase 1

Phase 1 was meant to simulate the immediate stage after the catastrophe that caused the global catastrophic infrastructure loss. phase 1 assumes the following:

1. No irrigation reliant on electrical pumps.
2. Full mechanization persists due to the availability of fuel.
3. Reduced input of fertilizers and pesticides due to the cessation of production, although remaining stocks were utilized.
4. Diminished availability of manure as animals were primarily slaughtered to prioritize food resources, retaining only those suitable for agricultural labor.

There should be enough fuel available to power agricultural machines for another year. The International Energy Agency set the annual demand of the agricultural industry in oil products at 111062 kt of oil equivalent (ktoe) in 2018.^[^ ^72^ ^]^ Available above‐ground fuel after a global catastrophic infrastructure loss was estimated at 319000 ktoe, encompassing 172000 ktoe of gasoline and 147000 ktoe of diesel.^[^ ^71^ ^]^ Considering that most agricultural machinery runs on diesel, the estimated stocks last for about a year while leaving the gasoline for critical transportation. Thus, the mechanization input dataset remains unchanged for phase 1.

Nitrogen (N) fertilizer application rates for phase 1 were calculated based on the annual global nitrogen surplus.^[^ ^73^ ^]^ This was done under the assumption that not all fertilizer that was produced was used in the same year. They project a surplus of 14477 kt N in 2020. In the first step, the amount of the nutrient applied in each cell was calculated as a fraction of the total amount of the nutrient summed over the crop‐specific harvested area with:

(4) $$N_{\mathrm{frac}}=\frac{N_{\mathrm{fert}}\times A_{\mathrm{crop}}}{\sum N_{\mathrm{fert}}\times A_{\mathrm{crop}}}$$

where N_fert_ is the application rate of the nutrient in kg ha^−1^ cell^−1^ and A_crop_ is the crop‐specific harvested area in ha cell^−1^. Each 5 arcminute cell had a specific application rate for N and a specific harvested area for each crop. The application rate was multiplied by the amount of crop area in each cell to determine the total amount of N applied to that cell. Then, this total was divided by the overall amount of N applied worldwide (the sum of N applied in all cells).

This division gives a fraction, which represents the proportion of N applied to the entire world that each cell receives. In the first phase, when only a reduced amount of N was available, this reduction applies equally to each cell. So, if each cell used to apply 100 units of N under normal conditions, during phase 1, they would only be able to apply 10 units of N because of the 90% reduction.

Then, the new total amount of the nutrient was calculated available for the specific crop N_total, crop_ in phase 1 based on the surplus reported by the FAO (2017).

(5) $$N_{\mathrm{total},\;\mathrm{crop}}=\frac{\sum N_{\mathrm{fert}}\times A_{\mathrm{crop}}}{T_{\mathrm{NG}}}\times T_{\mathrm{NG}1}$$

where *T*_NG_ is the total amount of the nutrient (NG = nutrient global) projected to be used for crop fertilization in 2020 and *T*_NG1_ is the projected nutrient surplus in 2020. The total amount of *N* used for crop fertilization is projected to be 118763 kt (FAO, 2017). Lastly, the new total is allocated back to the cells based on N_frac_:

(6) $$N_{\mathrm{fert},1}=\frac{N_{\mathrm{total},\;\mathrm{crop}}\times N_{\mathrm{frac}}}{A_{\mathrm{crop}}}$$

The pesticide application rates for phase 1 were calculated with the same approach as the fertilizer application rates. However, no data were available on the production surplus of pesticides generated in one year. Therefore, it was assumed that the surplus share of global pesticide production was in the same range as the share of the nutrients surplus in global nutrient production (≈10%). Equations (4) and (6) were formulated accordingly for pesticides but remained structurally the same. The new total of pesticides PE_total, crop_ available for a specific crop in phase 1 was calculated as follows:

(7) $$PE_{\mathrm{total},\;\mathrm{crop}}=\frac{\sum PE\times A_{\mathrm{crop}}}{T_{\mathrm{PEG}}}\times T_{\mathrm{PEG}}\times \frac{\frac{T_{\mathrm{nG}1}}{T_{\mathrm{nG}}}}{2}$$

where PE is the pesticide application rate in kg ha^−1^ cell^−1^, T_PEG_ is the total amount of pesticides used (PEG = pesticides global) for agricultural purposes in 2019,^[^ ^74^ ^]^ and *T*_nG1_ and *T*_nG_ referring to the totals defined above for nitrogen.

#### 2.4.2 Phase 2

In phase 2 all stocks were assumed to be depleted, hence, mechanisation_2_, n_fert2,_ and PE_2_ were set to zero. Manure application rates were expected to be the same for phases 1 and 2 as they were dependent on the available livestock. It was assumed that the human population would switch to a mostly vegan diet to use the calories that could be produced in the most efficient way possible. Therefore, only draft animals like horses, buffaloes, and cattle would be kept and fed on agricultural residues and roughage. For this analysis, only cattle was considered, as horses and buffalos only constitute a small fraction of global livestock and were not considered in the datasets available.^[^ ^63^ ^]^ To calculate new manure application rates, the labor demand in each grid cell was assessed in terms of needed cattle per grid cell by dividing the harvested area in each cell by the area that could be worked by one head of cattle (ha per head of cattle), which was assumed to be 7.4 ha per draft animal as a typical working capacity.^[^ ^75^ ^]^ Considering that modern cattle were not bred to work, this value could be expected to be considerably lower. To be conservative in terms of manure availability, 5 hectares per head of cattle was used. Next, the excretion rate of one head of cattle was calculated. In the manure dataset^[^ ^63^ ^]^ the total amount of manure produced in 2014, which amounts to 131000 kt N and the share of the manure produced by cattle, namely 43.7%. There were 1.44 billion head of cattle in 2014.^[^ ^76^ ^]^ Multiplying the total amount of manure with the fraction attributed to cattle and dividing the result by the heads of cattle in that year rendered an excretion rate of ≈40 kg N head^−1^ yr^−1^. In the last step, the new crop‐specific N manure application rate M_n, crop_ was computed by

(8) $$M_{n,\;\mathrm{crop}}=\frac{39.77\times C_{\mathrm{crop}}}{A_{\mathrm{crop}}}$$

where *C*_crop_ is the crop‐specific number of cattle in each grid cell. This means that the available manure comes from the draft cattle needed to labor the area in that cell.

For phase 1 M_n, crop_ was combined with n_fert 1_ into n_tot 1_. In phase 2 the N from manure was the only source of N left, so it was taken as the sole input.

As with manure, irrigation as a fraction of the cropland in a cell which was actually irrigated could not profit from first‐year stocks and therefore the same values were used for phase 1 and phase 2. A sharp reduction in actually irrigated areas was expected as large parts of the irrigation infrastructure were dependent on electricity and fossil fuels. Today, ≈20% of cultivated land was irrigated and it contributes 40% of global food production. To obtain the fraction of irrigated area, which was reliant on electricity, the information on the source of the irrigation water (surface or groundwater or other) was combined with country‐level statistics. The fraction of actually irrigated cropland in a global catastrophic infrastructure loss (GCIL) scenario I_gcil_ was calculated as follows:

(9) $$I_{\mathrm{gcil}}=I_{\mathrm{AC}}\times \left(1-I_{\mathrm{RC}}\right)$$

where *I*_AC_ is the total currently (AC = all currently) irrigated fraction of cropland in each cell and I_RC_ is the fraction of currently irrigated area which is reliant (RC = reliant currently) on electricity or diesel in each cell.

The datasets comprising the input variables for phases 1 and 2 were fed into the model specified above to predict the crop‐specific yields under global catastrophic infrastructure loss conditions. The predicted values were used to calculate the crop‐specific relative change in yield RC_C_ for each cell:

(10) $$RC_{\mathrm{crop}}=\frac{\left(Y_{\mathrm{PC}}-Y_{\mathrm{crop}}\right)}{Y_{\mathrm{crop}}}$$

where Y_PC_ is the predicted crop‐specific (PC) yield in the respective phase 1 or 2 and Y_Crop_ is the crop‐specific yield around 2010 taken from the SPAM2010 dataset. Values above zero, resulting from the generalized linear model, were set to zero as yield increase in a global catastrophic infrastructure loss scenario was not realistic. Rather, the positive values were taken as an indication for stable yields unaffected by catastrophic circumstances. For the predicted yield and relative change, descriptive statistics measures were computed for each phase and crop, namely the range of values, the total crop production, the weighted mean, and the corresponding confidence interval. The weighted mean was also calculated for each continent. The yield was weighted according to the corresponding harvested area while the relative change was weighted according to the crop production in 2010. The results of and additional information on these calculations can be found in reports/Report_descriptions.pdf and reports/Prediction_statistics.xlsx in the repository of this paper^[^ ^56^ ^]^).

## 3 Results

### 3.1 Model Calibration and Validation

A generalized linear model based on a gamma distribution with a log link was fitted for all crops using the same set of variables. The final model for each crop incorporated the explanatory variables listed in **Table** **2**. Most coefficients had, as anticipated, a positive impact on the expected yield, but the model struggled to accurately capture low yield values. Nearly all coefficients were statistically significant at a 5% significance level, except for three instances: In the wheat model, the thermal regime level 2 was not significantly different from level 1 and the moisture regime level 3 was not significantly different from level 2; in the soybean model, the nitrogen input did not have a significant impact. For soybean, nitrogen application was not a significant yield influencing factor as it is a leguminous plant that is able to fix nitrogen. Wheat is not a crop that is routinely grown under tropical conditions. Therefore, it is reasonable that the different tropical climates (T1 + T2, M2 + M3) result in similar yields and do not show significant differences from each other. Further, the thermal and moisture regime levels were combined due to low numbers of data points in extreme climates. However, the same number of levels was used for all crops to ensure model comparability between crops. Consequently, it does not reflect the ideal number of levels for each individual crop: for wheat, for example, the number of observations in T1 and T2 was very low, so they could have been combined into one class. Nonetheless, the separation was maintained to ensure consistency with the models for corn, rice, and soybean.

**Table 2:** List of Independent Variables Used in the Generalized Linear Model.

Variable	Description	Categorical/ Continuous	Unit/Categories
n_total	Total nitrogen input (includes fertilzer and manure input)	Continuous	Kg ha^−1^
pesticides	Cumulated pesticide input (contains 20 different substances, see Table 1)	Continuous	kgha^−1^
irrigation_tot	Fraction of irrigated cropland per cell	Continuous	Unitless, values between 0 and 1
mechanized	Use of agricultural machinery for farming activities	Categorical	0 = not mechanized; 1 = mechanized
thz_class	Thermal regime class	Categorical, dummy‐coded	T1 = Tropics, lowland; T2 = Tropics, highland; T3 = Subtropics, warm; T4 = Subtropics, moderately cool; T5 = Subtropics, cool; T6 = Temperate, moderate; T7 = Temperate, cool, Boreal + Arctic
mst_class	Moisture regime class	Categorical, dummy‐coded	M2 = Length of Growing Period(LGP) < 120 days; M3 = LGP 120–180 days, M4 = LGP 180–225 days; M5 = LGP 225–270 days; M6 = LGP > 270 days
soil_class	Soil/terrain‐related class	Categorical, dummy‐coded	S1 = Dominantly very steep terrain; S2 = Dominantly hydromorphic soils; S3 = No or few soil/terrain limitations; S4 = Moderate soil/terrain limitations; S5 = Severe soil/terrain limitations; L3 = Irrigated soils

**Figure 1:**
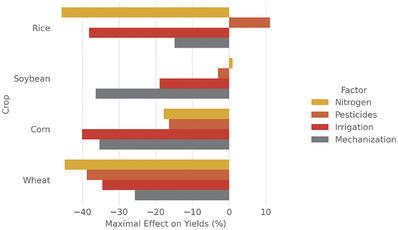
Projected yield change based on the difference between the maximal and minimal value for all factors by crop. Colors indicate the crop yield influence factor. This describes the maximum effect a single factor could possibly have, when the other values are held constant.

We measured the total yield change per factor by comparing the minimum and maximum input values while keeping other factors constant (see sheet YieldReductionPerFactor in reports/Model_results.xlsx) (**Figure** **1**). This difference was expressed as a percentage of the maximum input's yield, indicating the extent of yield change when the respective factor was absent. The most influential factor varied with the crop type. For corn, irrigation caused a notable 40% yield decrease. Total nitrogen application rate had the largest impact on rice and wheat yields, resulting in a 45% reduction. In contrast, soybean yield was most affected by the use of machinery, with a 36% decrease. Pesticide application had the lowest effect, notably impacting only wheat yields with a 39% reduction. Interestingly, rice yields showed an unexpected relationship with pesticide application. The model estimated a yield increase of over 10% when no pesticides were used (this is discussed in Chapter 4.1). Overall, irrigation had the most substantial negative impact on yields for three crops, followed closely by the use of agricultural machinery. Nitrogen application had a varying impact, causing the highest reduction for wheat and rice, while its effect on rice was relatively low (18% decrease) and negligible for soybean.

To calibrate the models, 80% of the data points were used, while the remaining 20% were reserved for validating the model fit using McFadden's *ρ* ^2^. The validated *ρ* ^2^‐values exhibited strong variation across different crops, with the highest agreement between data and model found for corn, yielding a *ρ* ^2^ of 0.47. The generalized linear model for rice achieved a *ρ* ^2^ of 0.40, while the wheat model obtained 0.36, and the lowest value was observed for soybean at 0.32. Nonetheless, all validation values indicated a good fit of the models to the data, as *ρ* ^2^ values ranging from 0.2 to 0.4 represent an excellent fit.^[^ ^77^ ^]^

The detailed model results for each crop including a 95% confidence interval for the coefficients and the corresponding goodness of fit metrics can be accessed in reports/Model_results.xlsx in the repository of this paper.)^[^ ^56^ ^]^

### 3.2 Mean Predicted Yield and Average Yield Reduction in a Global Catastrophic Infrastructure Loss Scenario

**Figure 2:**
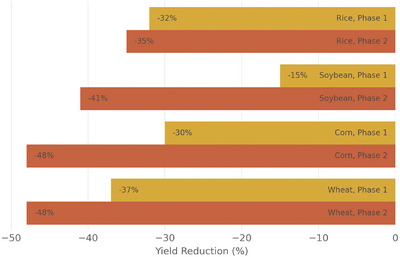
Projected yield reduction for phase 1 (some industrial inputs) and 2 (no industrial inputs) by crop. Values are weighted by the production of the cells (area times yield), as those areas are more important for food security. Colors indicate the phase.

**Figure 3:**
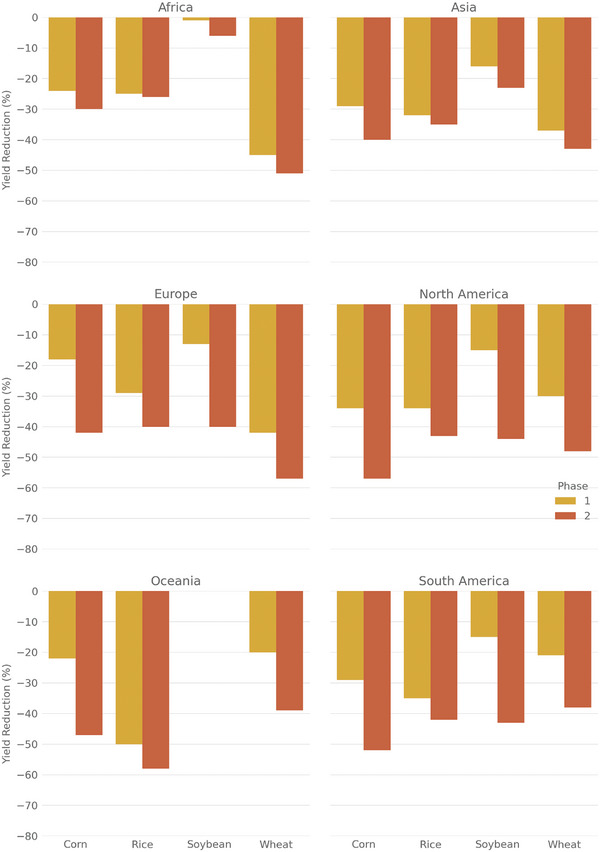
Projected yield reduction for phase 1 (some industrial inputs) and 2 (no industrial inputs) and all crops by continent. Values are weighted by the production of the cells (area times yield), as those areas are more important for food security. Colors indicate the phase.

The predicted yields show significant variation between phases 1 and 2, as well as across different crops and continents (**Figure** **2**,**3**). In Phase 1, the average reduction by the crop is between 15% and 37%, while in Phase 2, it increases to values between 35% and 48% (Figure 2). Among all the crops, soybeans experience the smallest reduction overall, especially in phase 1. The reductions differ greatly between phases 1 and 2 for all crops except rice. Rice yield reduction increases from 32% in phase 1 to 35% in phase 2. In contrast, soybeans perform relatively well in phase 1 but experience a large decrease in phase 2 (from 15% to 42% yield reduction). Both wheat and corn already exhibit substantial yield reductions in phase 1 (37% and 30% respectively), which further worsen in phase 2 (48% for both).

The magnitude of yield decrease also varies significantly by continent (Figure 3). Africa has the lowest average yield reduction, ≈26% over both phases, with little difference between the phases. Asia also shows a small disparity between phases 1 and 2, but the average yield reduction over both phases is at 32% notably higher compared to Africa. The difference between Phases 1 and 2 is more pronounced in the remaining continents where yield decreases by at least two‐thirds from Phase 1 to phase 2. Europe and South America face a similar reduction of ≈25% in phase 1 and 44% in Phase 2. With a projected decrease in yield of ≈30% in Phase 1 and almost 48% in Phase 2, North America and Oceania are most severely affected.

The detailed prediction results for each crop, phase and continent and, for comparison, also the metrics for the yield under current conditions are provided in reports/Prediction_statistics.xlsx in the repository of this paper.^[^ ^56^ ^]^ For further information on all plots presented in this work and their accompanying metrics, reports/Reports_descriptions can be consulted.

### 3.3 Spatial Patterns of Yield Loss

**Figure 4:**
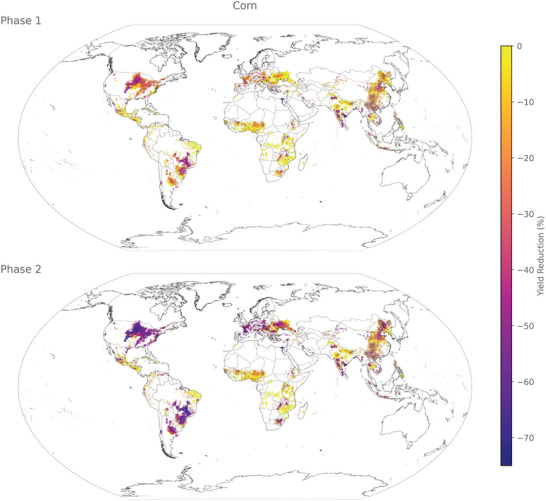
Spatial distribution of yield loss for corn in phases 1 (some industrial inputs) and 2 (no industrial inputs) at a resolution of 5 arcmin.

**Figure 5:**
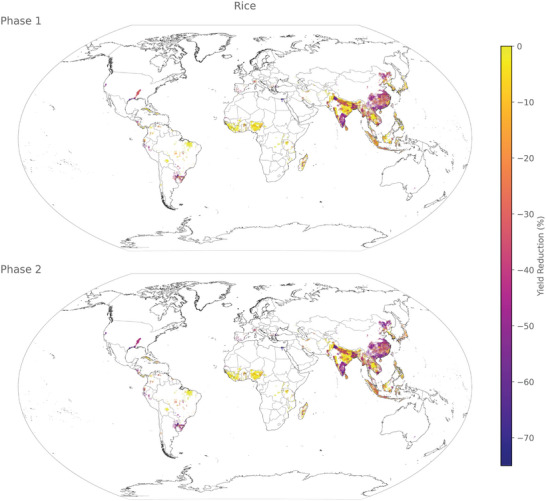
Spatial distribution of yield loss for rice in phases 1 (some industrial inputs) and 2 (no industrial inputs) at a resolution of 5 arcmin.

**Figure 6:**
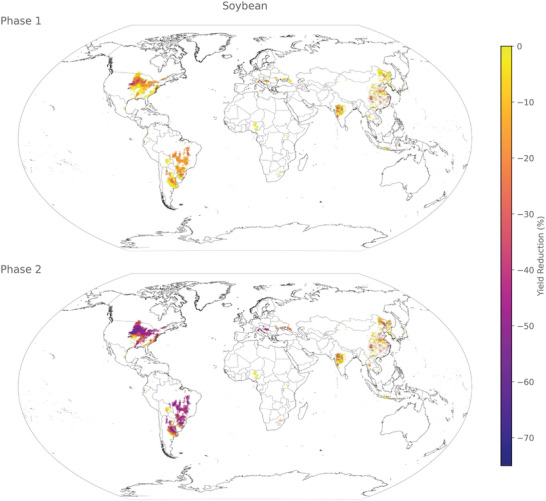
Spatial distribution of yield loss for soybean in phases 1 (some industrial inputs) and 2 (no industrial inputs) at a resolution of 5 arcmin.

**Figure 7:**
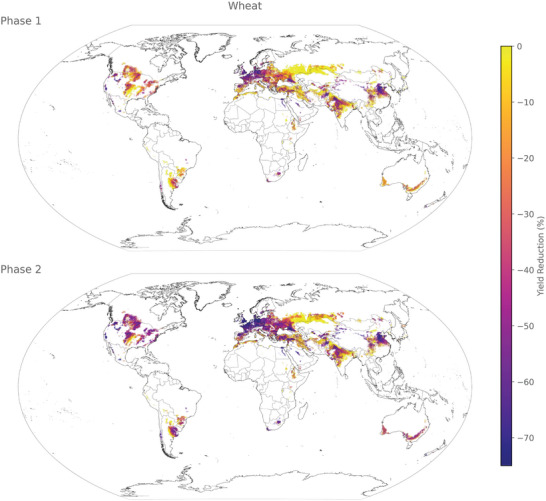
Spatial distribution of yield loss for wheat in phases 1 (some industrial inputs) and 2 (no industrial inputs) at a resolution of 5 arcmin.

The predicted yield loss reveals distinct hotspots in corn (**Figure** **4**), rice (**Figure** **5**), soybean (**Figure** **6**), and wheat (**Figure** **7**). The severity of the impact is amplified in phase 2, as the full repercussions of losing industrial inputs are felt. The modeled consequences to this impact are notably diverse, with regions showing heterogeneous patterns between pronounced and minimal effects. This mirrors the heterogeneous distribution of small‐scale and large‐scale agriculture in these areas today. When we consider the combined implications of these maps, it becomes evident that significant agricultural regions, like Central Europe, are anticipated to experience a substantial decrease of up to 75% in their potential production of rice, wheat, soybean, and corn. It seems likely that other crops, not modeled here, could see similar reductions. This means the major growing regions would experience a massive food shock, as they could lose the majority of their food production in 1–2 years. These reductions closely correlate with the current extent of industrialization in agriculture. Less intensively cultivated areas exhibit milder impacts, but they also tend to be less productive under current conditions.

#### 3.3.1 Corn

The effects on corn production vary considerably between phase 1 and phase 2. In phase 1, only a limited number of regions witness significant reductions in crop yields, primarily in southern India, southern Brazil, the Nile region, and the central USA. During phase 2, these regions experience even more pronounced declines in yield. Additionally, we observe a substantial drop in corn yield during phase 2 in Argentina, South Africa, Central Europe, Ukraine, the Balkans, and northern China.

#### 3.3.2 Rice

The geographical pattern of impacts remains highly consistent between Phase 1 and phase 2. The most severely affected areas include Southern Brazil, the Mississippi region, Southern India, the majority of China, and South‐East Asia, as well as some smaller regions where rice is cultivated in Europe and South America.

#### 3.3.3 Soybean

Similar to corn, soybean displays a significant contrast in its response between phase 1 and phase 2. In phase 1, substantial yield reductions are observed in only a few areas, primarily in the central USA and southeastern China. However, in phase 2, these affected regions expand significantly, encompassing most of the growing areas of the USA, Brazil, and Argentina, and the soybean cultivation in Europe, such as Austria.

#### 3.3.4 Wheat

Wheat encounters a substantial decrease in yield during phase 1, particularly in the western USA, certain areas of Argentina, the majority of Central Europe, India, and China. However, this worldwide decline in production worsens in phase 2, with significant yield reductions occurring in all wheat‐growing regions except for the Mississippi region in the USA, specific areas in South America, and Central Asia. This highlights both the general importance of wheat, as well as how strongly wheat yield is affected by the loss of the considered inputs.

## 4 Discussion

Following the first evaluations of the possible effects of a global catastrophic infrastructure loss scenario on agriculture^[^ ^31^ ^]^ this work proposes a formal modeling approach to investigate the issue, adds a spatial component to the analysis, and examines global catastrophic infrastructure loss consequences on agriculture in two different phases. Earlier research based on analyzing existing literature estimated pre‐industrial agricultural yield in a global catastrophic infrastructure loss scenario, which corresponds to a 60% drop from current yield levels.^[^ ^31^ ^]^ The modeled results here suggest that overall yields would drop by ≈35%–48% depending on the crop in phase 2, with corn and wheat (−48% in phase 2) experiencing the largest reduction., Areas with highly industrialized agriculture are affected much more severely and local yield reductions can reach 75% or more. However, yield reduction after a catastrophe would likely be larger than our estimate due to the lack of available data some relevant factors (e.g., cultivars or seed availability) could not be taken into account and most of the omitted factors would likely decrease yield even more. Still, the general trends visible in the prediction results are reliable and can be used as a guideline going forward. They show that a scenario as described here would be the largest threat to global food security which modern civilization has experienced and that preparation is needed to avoid such a catastrophe.

### 4.1 Implications of a Global Catastrophic Infrastructure Loss Scenario

#### 4.1.1 General Implications

The results demonstrate a substantial difference between phase 1 and 2 yield losses. It shows that Phase 1 can be critical in the preparation for Phase 2 because the yield losses are more manageable in the first phase. This can provide the time necessary to adapt to the new circumstances by building up non‐electrical logistic infrastructure, building tools and wagons, establishing a communication system, implementing new farming techniques and crop rotations to manage pests and nutrients, and overall adjusting as a society. The crucial component is the continued use of agricultural machinery as it ensures that tasks can be completed on large farms even as the preparations for the transition to a human and animal‐operated system are still underway. Furthermore, the evolving global conditions will significantly shape the eventual outcome of this situation. The persistent challenges, often referred to as the “polycrisis,” entail simultaneous stress on various systems.^[^ ^78^ ^]^ This encompasses issues such as planetary boundaries,^[^ ^26^ ^]^ the destabilization of political landscapes, both within nations and in international relations, and their impact on the food system^[^ ^79^ ^]^ and possible interactions between global disruptive events.^[^ ^80^ ^]^ The more severe this polycrisis becomes, the greater the challenge will be to adapt to a worldwide catastrophic infrastructure breakdown as outlined here, since addressing sudden and global crises requires global cooperation to be effective.^[^ ^81^ ^]^

#### 4.1.2 Global or Regional Catastrophic Infrastructure Loss

One crucial factor to consider is the magnitude of infrastructure loss. Various potential causes of infrastructure damage can vary significantly in their scale. Additionally, the impact of these factors can differ based on the geographic region. For instance, in the case of a nuclear war‐induced High‐Altitude Electromagnetic Pulse (HEMP), its effects are primarily concentrated on the countries involved in the conflict and their neighboring nations.^[^ ^34^ ^]^ Similarly, geomagnetic storms tend to affect specific regions, unless they reach very high magnitudes.^[^ ^37^, ^82^ ^]^ It is worth noting that even regional disruptions can have profound repercussions. Research has demonstrated that a substantial disturbance in food production, centered around a major producer on a continental scale, can have far‐reaching global consequences.^[^ ^2^, ^83^ ^]^ Furthermore, studies in natural hazards have revealed that such disruptions often serve as the initial point of a risk cascade, spreading impacts to areas and systems that were initially unaffected by the shock.^[^ ^84^ ^]^

We center our attention on global effects, as this allows an assessment of worst‐case scenarios where a global disruption impacts all regions simultaneously. Nonetheless, our analysis highlights specific regions particularly susceptible to catastrophic infrastructure loss on a regional scale as well. For instance, the breadbasket of Central Europe or the USA would be very vulnerable. In the event of a more regional catastrophe, these regions would pose the greatest risk of triggering a global cascade of adverse effects, as they are key players in global food trade now.

#### 4.1.3 Potential Countermeasures

While the research shows that a global catastrophic infrastructure loss scenario could potentially be devastating, this does not mean that nothing can be done to prevent or mitigate the effects. Based on our research and the existing literature, we have identified a range of potential countermeasures.

Our findings clearly indicate that agricultural regions with reduced dependence on inputs such as fertilizers and pesticides are less affected by infrastructure loss. This implies that incorporating more diverse agricultural practices aimed at reducing this dependency would enhance the resilience of the food system, a perspective in line with existing literature.^[^ ^1^ ^]^ Transitioning to approaches like smallholder farming, organic farming, or sustainable practices such as permaculture could be beneficial. However, it is important to acknowledge that these changes involve trade‐offs. Smallholder farming may render regions less resilient to local disruptions,^[^ ^22^ ^]^ organic farming may require larger land areas per unit of food production,^[^ ^85^ ^]^ and permaculture may demand substantial manual labor.^[^ ^86^ ^]^ Thus, it is important to ensure that in seeking solutions, humanity does not replace one problem with another.

An alternative path toward enhancing the resilience of food production involves investigating resilient food sources that remain viable even in the event of an electrical grid failure. Early research indicates the potential of various low‐tech solutions, currently underutilized, such as nutrient extraction from leaves.^[^ ^71^ ^]^

The primary adverse effects of global catastrophic infrastructure loss result from damage to the electrical grid. If the power supply fails, all other strategies for coping with a catastrophe become more challenging. The most direct approach to mitigate adverse consequences is to enhance the resilience of the physical infrastructure of the electrical grid against such incidents. Some governments have already initiated steps in this direction, as exemplified by the Obama administration's directive to federal agencies and departments to coordinate their preparations and responses to severe space weather events.^[^ ^82^ ^]^ There is still considerable work to be undertaken, and such measures may offer limited assistance if the infrastructure is compromised due to a cyber attack or insufficient maintenance during a major pandemic. Still, a robust infrastructure has been identified, as one of the factors that may make collapse less likely.^[^ ^87^ ^]^

There are certain threats to the power grid that can be anticipated, allowing for a period of forewarning before their actual impact. This timeframe offers us the opportunity to proactively fortify the grid and avert potential harm. Enhancing this warning period would increase the likelihood of effective preparations. For instance, when considering solar flares, the most significant ones necessitate the presence of a sunspot covering ≈10% of the solar surface. Detecting such a massive sunspot early on is likely feasible.^[^ ^38^ ^]^

For larger regional incidents and less severe global events, storage can serve as a viable option to bolster resilience. It offers an additional window of opportunity for electrical grid restoration and food production recovery. Nonetheless, the global food reserves typically last a mere 4–7 months.^[^ ^15^ ^]^ While increased food storage can act as an emergency measure, it comes at a high cost, potentially driving up global food prices and worsening current food insecurity. Furthermore, numerous catastrophic scenarios necessitate provisions for several years, posing substantial challenges in safely stockpiling such extensive quantities over extended durations.

Right now, the trade system exhibits vulnerability, as it is primarily centered around a handful of main trading hubs, such as the United States.^[^ ^1^, ^2^ ^]^ This concentration renders it vulnerable to disruptions in the event of a major hub failure. Numerous studies emphasize the potential for a rapid disintegration of the global food trade under such circumstances.^[^ ^2^, ^5^, ^48^ ^]^ The mitigation of this risk factor could be achieved through a more equal distribution of food trade among nations. The same also holds true for the number of companies in the food sector. Right now the food system is dominated by few, but very big international corporations.^[^ ^88^ ^]^ Smaller, but more dispersed companies might fare better after global catastrophic infrastructure loss.

The safety of the food system is significantly shaped by how societies respond to disruptions(social amplification of risks),^[^ ^1^ ^]^ particularly in the context of food export bans. Such bans have the potential to set off a chain reaction, causing countries to halt their exports out of fear that they will not be able to secure imports in return. This disruption could result in food insecurity, even when enough food still exists.^[^ ^83^, ^89^ ^]^ Another potential social consequence of the disruption of the food system is civil unrest, which could exacerbate problems.^[^ ^89^ ^]^ Pre‐established agreements between nations and emergency plans in countries on how to address such scenarios could enhance the likelihood of better outcomes.

### 4.2 Limitations

Our results provide the first spatially explicit estimate of the effects of a global catastrophic infrastructure loss scenario on crop yield. The spatial pattern of yield loss corresponds with the anticipation that highly industrialized agriculture would be most severely affected. Further refinement of these findings is recommended for future research. On a global scale, achieving a better estimate is mainly limited by the availability of new, more accurate datasets. Hence, this study likely offers an accurate global estimate attainable with current data.

#### 4.2.1 Limitations in the Available Data

The datasets do not directly reflect the true distribution of specific variables but instead offer a statistical approximation obtained through downsampling. This introduces uncertainties that are consequently mirrored in our model's output.

The datasets used in this analysis are not harmonized, only some applied standardization against country‐level FAOSTAT data. Consequently, the layers do not perfectly align, differing in both their extent and spatial distribution. These discrepancies in extent result in missing data points within the consolidated dataset used in the analysis. Notably, the mechanized and pesticide datasets cover significantly fewer cells than others, particularly in Africa. This necessitated the removal of many cells before calibrating the model, especially in Africa, as some of the data is more uncertain there^[^ ^57^ ^]^ (Additional information on the data cleaning process and the effect of each operation on the metrics of the datasets can be found in reports/Report_descriptions.pdf and reports/Descriptive_statistics.xlsx in the repository of this paper^[^ ^56^ ^]^). Despite the exclusion of many cells during model calibration, the remaining data still largely represent the main growing regions and the majority of annual crop production for each crop.

Due to limited data availability, some factors, which are essential for estimating yields in a scenario of global catastrophic infrastructure loss, were omitted from the generalized linear model. These factors include seed availability, the dominant crop varieties, and farmers' knowledge, accessibility of feed for draft animals, tools and materials for agricultural work, the health of draft animals, population displacement, climatic changes, alternative pest control methods, crop rotations, alternative sources of fertilizers, food preservation methods, and the time required for animal slaughtering. All these factors and aspects have the potential to either enhance or diminish crop yields in a scenario of global catastrophic infrastructure loss. However, most are likely to exacerbate the catastrophic impact.

Among these factors, the three most significant are seed availability, the dominant crop varieties, and farmers' adaptability to a significant shift in production techniques. Seed availability and the prevalence of specific crop varieties are closely intertwined. Many farmers, particularly in industrialized nations, purchase seeds from large global corporations rather than saving seeds from their own harvests. While this practice can be altered if necessary, these varieties are often bred to excel under high‐input conditions and are designed for repurchase. This does not imply that these seeds will not grow or perform poorly under low‐input conditions, but they are more susceptible to crop failures compared to local landraces.^[^ ^90^ ^]^

In the event of a global failure of electrical infrastructure, highly specialized and industrialized plant breeding and seed production would likely be disrupted. Corn, in particular, would be severely affected, as the majority of corn crops are grown from hybrid seeds specifically engineered for high one‐year performance. If seeds from large companies become unavailable and saved seeds from high‐yielding varieties perform inadequately in the scenario of global catastrophic infrastructure loss, there may not be enough landrace seeds to cultivate the entire current cropland area.

Transitioning from highly mechanized agriculture to traditional farming methods could pose a challenge for many farmers. However, some small farms still employ traditional knowledge, serving as valuable resources for re‐educating farmers in these traditional techniques.

#### 4.2.2 Limitations in the Model

The fitted models face challenges in accurately capturing yields in areas where the yield is already very low today and tend to estimate a more moderate range of values than the training data, particularly for low yields. The minimum yield prediction by the model is higher than the observed minimal yield in the SPAM2010 yield dataset. It suggests that lower yields are only marginally, if at all, negatively affected by global catastrophic infrastructure loss. These are areas where very little external inputs like fertilizers are used today, which would make them less affected by infrastructure loss. Reasons for the inadequate model fit on lower yields could include data misalignment, the particularities of the selected link function, and the omission of relevant variables.

Notably, for rice and soybean, the models estimated a negative relationship between agricultural input and crop yield, which is unexpected. For soybeans, the negative effect of nitrogen application on crop yield is not statistically significant and is not a cause for concern, given soybean's ability to fix nitrogen from the air. However, the stronger, statistically significant negative effect of pesticide application on rice is surprising. The inaccurate mapping of the relationship between rice and pesticide application could possibly be influenced by data misalignment, data quality variations in different growing regions, and calibration on smaller units.

### 4.3 Recommendations for Future Research

Moving forward, future research should focus on:

- Enhancing the accuracy of the estimate by refining the statistical methodology used here, or by combining a statistical framework with machine learning methods, or process‐based crop models.
- Including data for missing factors such as seed availability. Additionally, data with the same resolution (5 arcmin in this study) should be collected instead of downsampling from country‐level data. By incorporating more precise and comprehensive datasets into this analysis, its accuracy could be improved.
- Exploring resilient food options that could serve as viable alternatives to conventional food production in the event of global catastrophic infrastructure loss. For instance, seaweed, which has demonstrated promise following other global catastrophes,^[^ ^47^ ^]^ may also prove beneficial in this context due to its ability to thrive in low‐tech cultivation. Also, leaf protein concentrate can be produced at the community scale.^[^ ^91^ ^]^
- Estimating the scale‐up capability of hand/animal tools, as well as wood chipping and gasification to provide fuel for equipment. This paper assumes that the production and distribution of such tools are possible. Without these tools, yield losses would be higher.
- Investigating backup communication systems to facilitate coordination and production of food and other necessities after the catastrophe.^[^ ^42^ ^]^
- Developing comprehensive disaster‐specific preparedness and response plans for each country. This includes identifying potential food sources, determining the optimal regions for cultivation, and optimizing food distribution strategies to ensure the nutritional needs of all citizens are met. Such a plan has already been created for Argentina in preparation for a nuclear winter.^[^ ^92^ ^]^
- Analyzing potential yield loss for different food crops. The crops outlined in this study account for ≈60% of the total food required for human consumption, it remains relevant to investigate the fate of the remaining 40%.
- Gathering insights on the response of the economic system to a global catastrophe. The research presented in this context operates under the assumption that global trade largely ceases due to the unavailability of transportation means, while the distribution of fuel, fertilizer, and pesticides remains possible. The persistence of long‐distance trade networks could mitigate many of the challenges outlined earlier. Therefore, gaining insights into how the economy and trade might adapt becomes highly valuable, enabling the development of strategies and safeguards to facilitate trade even in the wake of a global catastrophe. There have been the first studies to understand agricultural economics after other global catastrophes like nuclear war^[^ ^93^ ^]^ and how climate change could change trade communities.^[^ ^29^ ^]^ Such models could potentially be adapted to the scenario explored here.
- Analyzing specific regions, especially in the identified hotspots and within Africa, could offer valuable insights. In the regions facing the most severe impacts, it might be beneficial to examine the results at the country level to provide recommendations at that scale.

## 5 Conclusion

The food supply chain faces significant vulnerability due to the potential for global catastrophic infrastructure loss. In this study, we have refined prior assessments by conducting a spatially explicit global analysis of the potential reduction in crop yields resulting from the loss of essential inputs such as nutrients, mechanization, irrigation, and pesticides. This analysis reveals that such an event would significantly disrupt food production. On average, we anticipate a roughly 40%–50% reduction in current crop yields when fertilizer and nutrient stocks are depleted. Regions with high levels of industrialization, such as Central Europe, may experience even more substantial declines. It is important to note that our assessment may underestimate the full extent of potential consequences, as we were unable to consider various critical factors like seed diversity, scale‐up capability of tools needed for less mechanized agriculture, and farmers' knowledge due to data limitations. Nevertheless, it is important to recognize that we do have options and can take action to address this challenge.

We have also identified a range of potential countermeasures, including the diversification of agricultural systems to reduce dependence on international trade and enhance local food self‐sufficiency. Implementing these measures is likely to enhance the resilience of the food system against the disruptions explored in this study. Furthermore, we have pinpointed areas for future research. Creating new datasets likely constitutes the most impactful step to improve model accuracy. Better data can help to bridge the knowledge gaps regarding missing factors and to gain a deeper understanding of how both, the global food trade and production systems as well as the economy, would react to such a substantial shock.

Finally, countries can enhance their resilience to the mentioned catastrophes by formulating preparedness and response plans. These plans should explore how a particular country can utilize its resources to adjust to post‐catastrophe conditions.

## Conflict of Interest

The authors declare no conflict of interest.

## Data Availability

The data that support the findings of this study are openly available in Data is available as specified in Table 1. at [DOI], reference number [1]. These data were derived from the following resources available in the public domain: [Resource 1]; [Resource 2].
